# Indocyanine Green Fluorescence-Guided Axillary Reverse Mapping for Axillary Lymph Node Dissection in Breast Cancer: The FARM Trial

**DOI:** 10.1245/s10434-025-17703-0

**Published:** 2025-06-23

**Authors:** Chu Luan Nguyen, Deepali Poels, Basilie Teoh, Pratik Rastogi, Jue Li Seah, Belinda Chan, Susannah Graham, Farhad Azimi, Cindy Mak, Carlo Pulitano, Sanjay Kumar Warrier

**Affiliations:** 1 Department of Breast Surgery, Chris O’Brien Lifehouse, Camperdown, Australia; 2 Department of Surgery, Royal Prince Alfred Hospital, Camperdown, Australia; 3 Department of Surgery, The University of Sydney, Camperdown, Australia; 4 Department of Plastic and Reconstructive Surgery, Chris O’Brien Lifehouse, Camperdown, Australia

**Keywords:** Indocyanine green, Fluorescence, Axillary reverse mapping, Axillary lymph node dissection, Breast cancer

## Abstract

**Background:**

Lymphedema is a significant complication following axillary lymph node dissection (ALND). Axillary reverse mapping (ARM) is a technique aimed at identifying and preserving lymphatic drainage of the upper limb during ALND to prevent lymphedema. However, concerns regarding tracer reliability and oncological safety have hindered its widespread use. This study assessed feasibility of indocyanine green fluorescence-guided ARM during ALND and explored predictive factors for ARM node metastasis.

**Methods:**

In this prospective trial (ACTRN12621000817842), patients with clinically node-positive breast cancer or positive sentinel lymph node biopsy requiring ALND (2022–2025) were enrolled. Indocyanine green was injected into the upper arm to visualize lymphatics using near-infrared fluorescence during ALND. Axillary reverse mapping nodes were categorized by anatomical zone based on intersection of lateral thoracic vein (vertical) and second intercostobrachial nerve (horizontal). Axillary reverse mapping nodes were sent separately for histopathological analysis. Univariate and multivariate analyses were performed on patient, tumor, and nodal characteristics.

**Results:**

Among 100 patients, ARM nodes were identified in 95% (95% confidence interval [CI] 88.7–98.4), yielding 111 nodes. Of these, 68.5% were located in the upper lateral axilla. Metastatic involvement of ARM nodes occurred in 18.9% of cases. Multivariate analysis identified tumor size ≥ 50 mm (hazard ratio [HR] 1.98, 95% confidence interval [CI] 0.25–3.83, *p* = 0.04) and higher nodal stage (N2/N3) (HR 3.04, 95% CI 1.37–4.58, *p* = 0.015) as independent predictors of ARM node metastasis.

**Conclusions:**

Indocyanine green fluorescence-guided ARM is a feasible technique during ALND. However, the risk of ARM node metastasis in advanced disease suggests that routine ARM node preservation may be unsafe, indicating the need for alternative strategies, such as lymphaticovenous anastomosis, to mitigate lymphedema risk.

Lymphedema remains a significant complication for breast cancer survivors following axillary surgery. Reported incidence rates vary considerably, up to 30%, depending on the diagnostic methods and criteria applied. This chronic condition can significantly impair quality of life, affecting body image, limiting social and occupational activities, and increasing healthcare costs.^1–3^ Although the use of axillary lymph node dissection (ALND) has declined with the paradigm shift towards de-escalation of the axilla, it remains an essential procedure for selected patients.

Axillary reverse mapping (ARM) is a technique developed to identify and potentially preserve lymphatic drainage pathways of the upper limb during ALND in an effort to reduce the risk of postoperative lymphedema.^4,5^ The technique involves injecting a tracer into the upper arm to visualize arm-draining lymphatics, similar to how sentinel lymph node (SLN) mapping is performed for breast cancer. Traditionally, ARM has relied on blue dye or radioisotopes, but these agents have shown variable reliability and accuracy. Reported detection rates for blue dye are 61–71% and lymphatic visualization is unreliable, with additional risks of skin staining and hypersensitivity reactions. Lymphoscintigraphy with technetium-99m offers enhanced node identification but lacks direct visualization of lymphatic vessels.^4,5^ To overcome these limitations, near-infrared fluorescence imaging with the green fluorophore, indocyanine green (ICG), has emerged as a promising alternative.^6–15^ When ICG is injected subdermal, lymphatics draining the upper limb glow brightly as they fluoresce under near-infrared light, providing the surgeon with a dynamic, real-time visualization of lymphatic pathways. Indocyanine green has a strong safety profile, and its peak emission at 820 nm minimizes background noise, enabling clear imaging of lymphatics and nodes.^16–18^

While early studies suggested ARM nodes were rarely involved with metastases, more recent evidence indicates that metastatic involvement is not uncommon.^4,5,12,19–23^ The combination of a significant rate of ARM node metastasis and limitations associated with traditional tracers has raised concerns about the oncological safety of preserving these nodes.^14,23^ This study aimed to evaluate the feasibility of ICG fluorescence-guided ARM during ALND, as well as predictive factors for ARM node metastasis.

## Methods

### Study Design and Participants

This prospective study enrolled female patients diagnosed with invasive breast cancer who were scheduled to undergo ALND between January 2022 and January 2025. Inclusion criteria were older than 18 years, and either core needle biopsy-confirmed clinically node-positive disease or SLN positivity in clinically node-negative patients not meeting the ACOSOG Z0011 criteria.1 Patients were excluded if they had distant metastases, inflammatory breast cancer, prior axillary surgery, or a known hypersensitivity to ICG. Ethics approval was granted by the institutional review board (Protocol No: X21-0142 & 2021/ETH00767), and the trial was registered with the Australian New Zealand Clinical Trials Registry (ACTRN12621000817842).

The primary aim of the study was to evaluate the feasibility of identifying ARM nodes using ICG fluorescence. The secondary aim was to identify predictive factors associated with ARM node metastases.

### Surgical Procedure

**Fig. 1:**
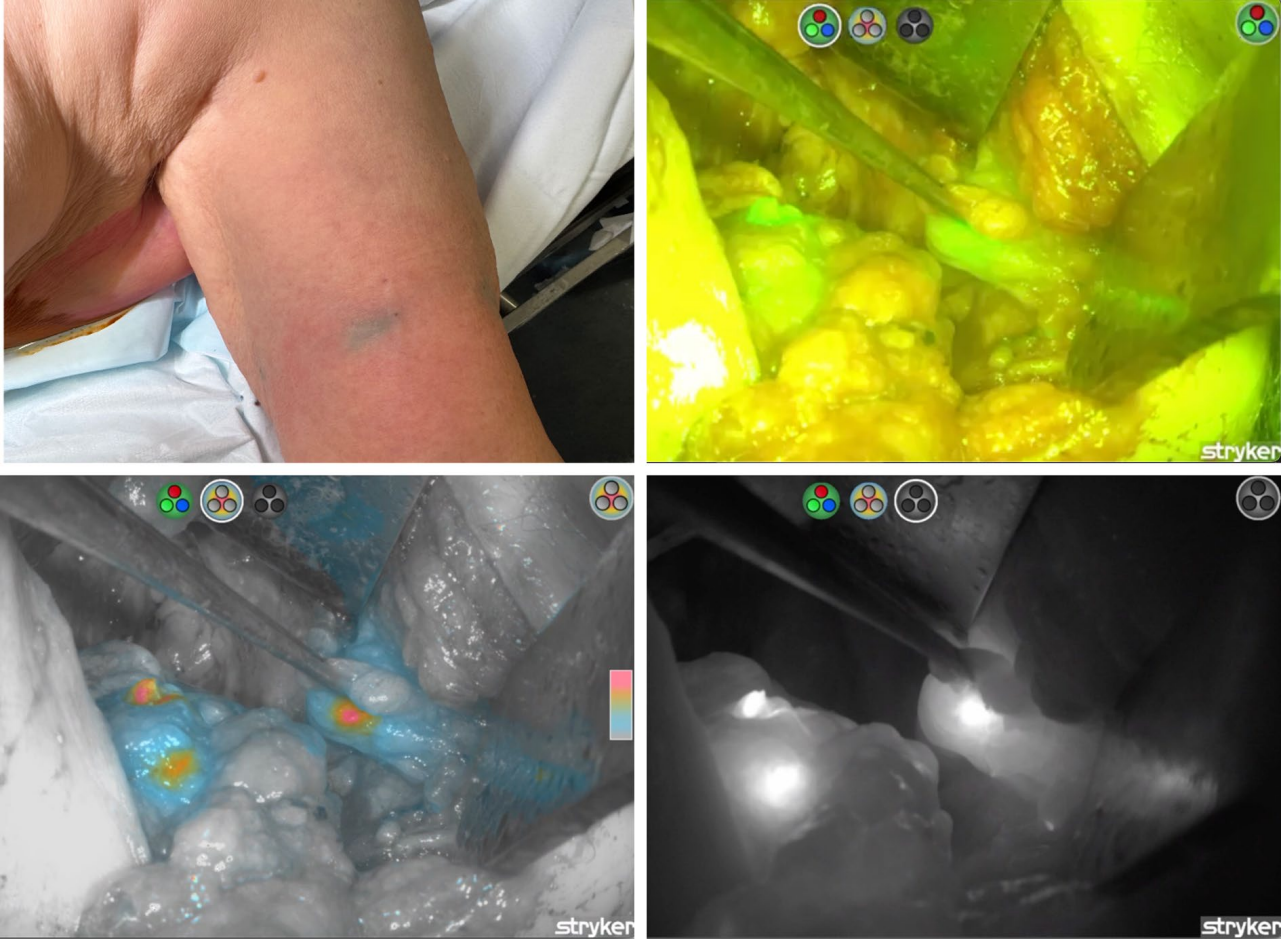
Left axillary lymph node dissection with ICG fluorescence-guided axillary reverse mapping. Top left image: 2 mL of ICG (5 mg/1 mL), divided into four 0.5-mL aliquots, injected subdermal into upper inner arm. Top right image: ARM nodes identified on “overlay fluorescence mode” with fluorescence image (green) displayed over a white light image. Bottom left image: ARM nodes observed in “colour segmented fluorescence mode.” White light image displayed in greyscale with fluorescence overlaid in a colour scale. Increasing intensities of fluorescence transition from blue to yellow to red. Bottom right image: ARM nodes observed in “fluorescence mode.” Fluorescence image displayed in greyscale, providing the highest level of contrast between fluoresced and non-fluoresced tissue. ARM: axillary reverse mapping

Significant prior experience with ICG fluorescence at this institution had been gained following its replacement of blue dye for SLN biopsy since 2021.^17,18^ For the ARM technique, after induction of general anaesthesia and before skin incision, 2 mL of Verdye^®^ (indocyanine green, 5 mg/1 mL, Diagnostic Green GmbH, Germany) was injected intradermally into the upper inner arm on the side of surgery. The injection was divided into four 0.5-mL aliquots administered around the arm. The area was massaged for 5 min to facilitate lymphatic uptake. Axillary reverse mapping node fluorescence was visualized using a near-infrared hand-held camera (SPY-PHI, Stryker Sydney, NSW, Australia), and displayed on a monitor (Fig. 1). Subsequently, standard mastectomy or breast-conserving surgery with ALND (levels I and II) was performed with the axillary dissection performed through the same incision or a separate incision if required.

**Fig. 2:**
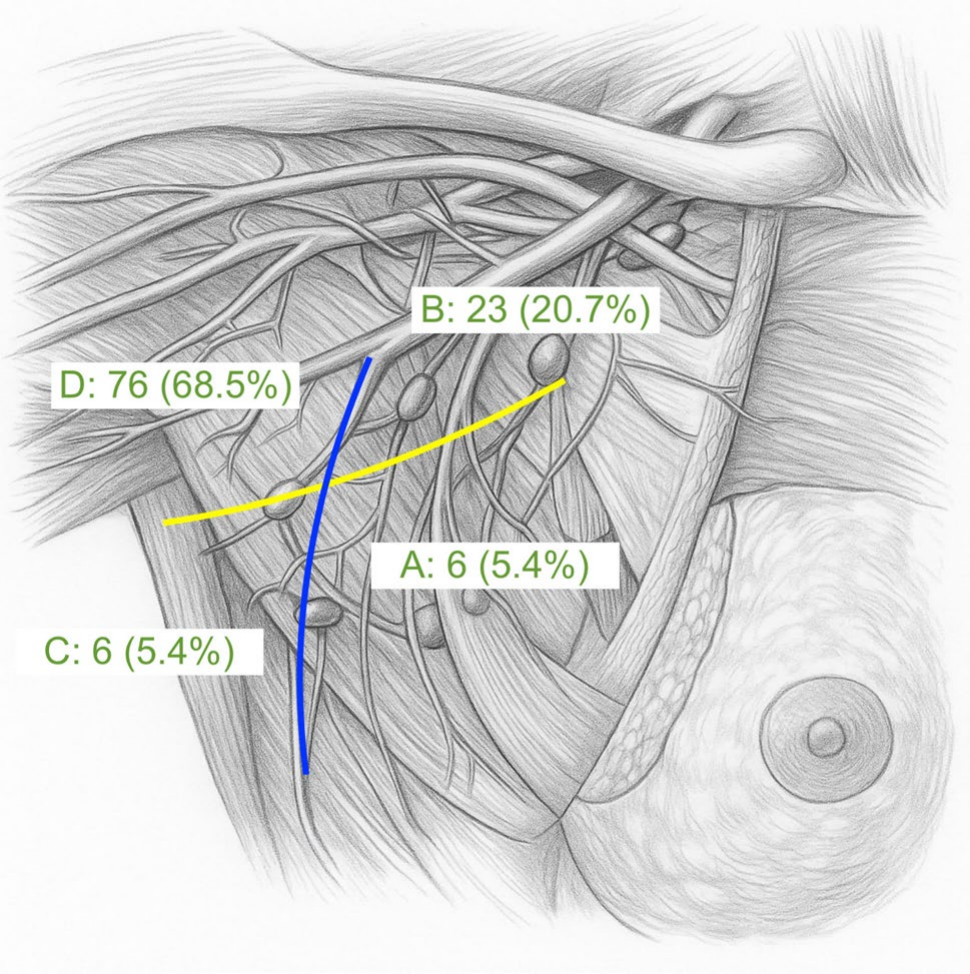
Location of ARM nodes mapped with ICG fluorescence. Axillary basin anatomically divided into four zones (**A–D**) by lateral thoracic vein (LTV) vertically and second intercostobrachial nerve (ICBN) horizontally. *ARM* axillary reverse mapping

Fluorescent ARM nodes were identified and excised (Fig. 1). Their location within the axilla was recorded using a four-zone anatomical map, divided by the lateral thoracic vein (vertical axis) and the second intercostobrachial nerve (horizontal axis), as described in previous studies (Fig. 2).^4,5,24^ Axillary reverse mapping nodes were separated from the remaining nodal tissue and sent for independent histopathological evaluation.

### Histopathological Assessment

All lymph nodes in ALND specimens were isolated, sliced in two parts equally, paraffin-embedded, and sectioned for hematoxylin-eosin-safran (HES) staining. For both ALND and ARM specimens, the total number of lymph nodes, number of metastatic nodes, and the size of metastases were recorded. Metastases were categorized as isolated tumor cells (<0.2 mm), micrometastases (0.2–2 mm), or macrometastases (>2 mm). ARM nodes with either micrometastases or macrometastases were classified as positive, while those with isolated tumor cells were classified as negative.

### Statistical Analysis

Data were reported as median with interquartile range (IQR) or mean with standard deviation (SD), as appropriate. Categorical variables were compared using chi-squared or Fisher’s exact test, and continuous variables using the Wilcoxon rank-sum test. Variables associated with ARM node metastasis were first evaluated with univariate analysis, followed by multivariable regression for covariates with a univariate *p* value ≤ 0.2. The Cox proportional hazards model was used for multivariate analysis. To estimate the detection rate of ARM nodes using ICG fluorescence, the sample size calculation was based on the expected proportion of successful visualization. Previous studies report detection rates ranging from 61 to 95%.^5,10,22^ A detection rate of 85% was conservatively assumed. Using a normal approximation to estimate a 95% confidence interval (CI) with a margin of error no greater than ± 7.5%, it was determined that a sample size of 100 patients would be sufficient to provide a precise estimate of the detection rate. A *p* value ≤ 0.05 was considered statistically significant. Analyses were conducted by using RStudio (version 2024.09).

## Results

### Patient Characteristics and ARM Node Detection

There were 115 patients initially enrolled and 15 of these were excluded as they had a targeted axillary dissection after becoming clinically node negative following neoadjuvant chemotherapy and avoided an ALND after obtaining a complete pathological response. A total of 100 patients undergoing ALND for invasive breast cancer were enrolled, with ICG fluorescence-guided ARM performed at the same time. ARM nodes were successfully identified in 95 of 100 patients (95%, 95% CI 88.7–98.4). Among these, a single ARM node was found in 81 patients (85.3%), two nodes in 12 patients (12.6%), and three nodes in two patients (2.1%), with a mean of 1.2 (SD 0.4) ARM nodes detected per patient.

For patients with at least one ARM node identified, the mean age was 52.6 years (SD 9.1) and mean body mass index (BMI) was 26.1 kg/m2 (SD 4.9). Tumor staging included T1 in 30 patients (31.6%), T2 in 50 (52.6%), T3 in 12 (12.6%), and T4 in 3 (3.2%). The majority of tumors were grade 3 (61.1%), estrogen receptor (ER) positive (64.2%), and progesterone receptor (PR) positive (62.1%). The proportion of HER2-positive cases was 23.2%, and for triple-negative it was 30.5%. Breast-conserving surgery was performed in 44.2% of patients, while the remainder underwent mastectomy. More than half of the cohort (58.9%) received neoadjuvant chemotherapy (Table 1).

**Table 1:** Patient and tumor characteristics in patients with and without ARM node mapped

Characteristic	ARM node mapped, *N* = 95	ARM node not mapped, *N* = 5	*p*
*Patient*			
Age, year, mean (SD)	52.6 (9.1)	52.8 (4.5)	0.959
BMI, kg/m^2^, mean (SD)	26.1 (4.9)	28.7 (2)	0.245
ASA, median (IQR)	2 (2, 2)	2 (2, 2)	1
Neoadjuvant chemotherapy, N (%)	56 (58.9)	2 (40)	0.71
*Tumor, n (%)*			
Type			0.126
No special type	81 (85.3)	4 (80)	
Invasive lobular	9 (9.5)	1 (20)	
Mixed	5 (5.2)	0	
Grade			0.852
1	5 (5.3)	0	
2	32 (33.7)	2 (40)	
3	58 (61.1)	3 (60)	
Clinical T stage			0.641
T1	30 (31.6)	3 (60)	
T2	50 (52.6)	1 (20)	
T3	12 (12.6)	1 (20)	
T4	3 (3.2)	0	
Clinical N stage			0.897
N0	36 (37.9)	2 (40)	
N1	49 (51.6)	3 (60)	
N2	6 (6.3)	0	
N3	4 (4.2)	0	
Receptor status			
ER+	61 (64.2)	4 (80)	0.809
PR+	59 (62.1)	4 (80)	0.739
HER2+	22 (23.2)	0	0.506
Ki67 index ≥ 14%	38 (40)	1 (20)	0.672
Triple negative	29 (30.5)	1 (20)	1
Breast operation, *n* (%)			0.818
Breast-conserving surgery	42 (44.2)	3 (60)	
Mastectomy	53 (55.8)	2 (40)	

*ARM* axillary reverse mapping, *SD* standard deviation, *IQR* interquartile range, *ASA* American Society of Anesthesiologists Classification, *BMI* body mass index, *ER+* estrogen receptor-positive, *PR+* progesterone receptor-positive, *HER2+* herceptin receptor-positive

Across these 95 patients, a total of 111 ARM nodes were identified. Most ARM nodes were located in anatomical zone D (*n* = 76, 68.5%), followed by zone B (*n* = 23, 20.7%), zone C (*n* = 6, 5.4%), and zone A (*n* = 6, 5.4%) (Table 2, Fig. 2). There were no adverse reactions to ICG, including allergic events, during surgery or in the immediate period one week postoperative.

**Table 2:** Characteristics of ARM nodes identified

	*N* = 95
*ARM nodes identified, n (%)*	
1	81 (85.3)
2	12 (12.6)
3	2 (2.1)
*ARM node characteristics*	
Total number of nodes identified	111
Mean number of nodes identified per patient (SD)	1.2 (0.4)
Zone of identified nodes, *n* (%)	
A	6 (5.4)
B	23 (20.7)
C	6 (5.4)
D	76 (68.5)
Metastatic ARM node, *n* (%)	18 (18.9)

*ARM* axillary reverse mapping, *SD* standard deviation

### Comparison of Patients with and Without Arm Node Detection

No significant differences in clinicopathological characteristics were observed between the 95 patients in whom ARM nodes were successfully identified and the five patients in whom they were not. Among those without ARM node detection, all but one had hormone receptor-positive tumors, with the remaining case being triple-negative (Table 1).

### Comparison Based on ARM Node Metastatic Status

Among the 95 patients with identifiable ARM nodes, 18 (18.9%) were found to have metastatic involvement. There was no significant difference in neoadjuvant chemotherapy completion between patients with positive ARM nodes and those with negative ARM nodes (*p* = 0.261). Compared to patients with negative ARM nodes, those with metastatic ARM nodes had a significantly higher incidence of clinical nodal stage N2/ N3 (38.9% vs. 3.9%, *p* < 0.001), larger mean tumor size (33.8 mm vs. 25.3 mm, *p* = 0.025), and a greater proportion of multifocal tumors (27.8% vs. 7.8%, *p* = 0.048) (Table 3). On multivariate analysis, independent predictors of metastatic involvement of ARM nodes included tumor size ≥ 50 mm (HR 1.98, 95% CI 0.25–3.83, *p* = 0.04) and higher clinical nodal stage (N2/ N3) (HR 3.04, 95% CI 1.37–4.58, *p* = 0.015) (Table 4).

**Table 3:** Clinical correlates of ARM nodes that were positive or negative for metastatic disease

	Positive ARM node, *N* = 18	Negative ARM node, *N* = 77	*p*
*Neoadjuvant chemotherapy, n (%)*			0.261
Yes	8 (44.4)	48 (62.3)	
No	10 (55.6)	29 (37.7)	
Nodal characteristics			
Clinically palpable node, *n* (%)			0.08
Yes	11 (61.1)	27 (35.1)	
No	7 (38.9)	50 (64.9)	
Clinical node status, *n* (%)			**< 0.001**
N0/N1	11 (61.1)	74 (96.1)	
N2/N3	7 (38.9)	3 (3.9)	
Total lymph nodes excised, mean (SD)	19.1 (4.5)	20.2 (5.6)	0.421
Number of ARM nodes mapped			0.21
< 2	12 (66.7)	69 (89.6)	
≥ 2	6 (33.3)	8 (10.4)	
*Tumor characteristics*			
Tumor type, *n* (%)			0.28
No special type	17 (94.4)	68 (88.3)	
Invasive lobular	1 (5.6)	9 (11.7)	
Tumor grade, n (%)			0.084
1/2	11 (61.1)	26 (33.8)	
3	7 (38.9)	51 (66.2)	
Tumor size, mm, mean (SD)	33.8 (16.7)	25.3 (13.5)	**0.025**
Multifocal, *n* (%)			**0.048**
Present	5 (27.8)	6 (7.8)	
Absent	13 (72.2)	71 (92.2)	
Lymphovascular invasion, *n* (%)			0.231
Present	13 (72.2)	41 (53.2)	
Absent	5 (27.8)	36 (46.8)	
*Hormonal status*			
ER status, *n* (%)			0.563
Positive	10 (55.6)	51 (66.2)	
Negative	8 (44.4)	26 (33.8)	
PR status, *n* (%)			1
Positive	12 (66.7)	53 (68.8)	
Negative	6 (33.3)	24 (31.2)	
HER2 enriched, *n* (%)			0.409
Present	6 (33.3)	16 (20.8)	
Absent	12 (66.7)	61 (79.2)	
Triple negative, *n* (%)			0.568
Present	7 (38.9)	22 (28.6)	
Absent	11 (61.1)	55 (71.4)	

Bold values indicate statistical significance (*p* ≤ 0.05)

*ARM* axillary reverse mapping, *SD* standard deviation, *IQR* interquartile range, *ASA* American Society of Anesthesiologists Classification, *BMI* body mass index, *ER+* estrogen receptor-positive, *PR+* progesterone receptor-positive, *HER2+* herceptin receptor-positive

**Table 4:** Predictors of metastatic ARM nodes

	Hazard ratio (95% CI)	*p*
Clinically palpable node		0.211
Absent	1	
Present	1.89 (0.7, 3.15)	
Clinical nodal status		**0.015**
N0/N1	1	
N2/N3	3.04 (1.37, 4.58)	
Tumor size (mm)		**0.04**
< 50	1	
≥ 50	1.98 (0.25, 3.83)	
Multifocal		0.661
Absent	1	
Present	1.29 (0.42, 2.95)	

Bold values indicate statistical significance (*p* ≤ 0.05)

*ARM* axillary reverse mapping, *CI* confidence interval, *HR* hazard ratio, HR <1: decreased odds of metastatic ARM node, HR >1: increased odds of metastatic ARM node

## Discussion

This study evaluated the utility of ICG fluorescence-guided ARM during ALND in breast cancer. In a cohort of 100 patients, the fluorescence-guided technique demonstrated a high ARM node detection rate of 95%. However, ARM nodes were involved with metastases in 18.9% of patients, particularly among those with larger tumors and greater axillary nodal burden, suggesting that ARM node preservation in such cases may be oncologically unsafe.

The detection and preservation of arm lymph nodes using the ARM technique is based on the theory that the lymphatic pathways of the upper limb and breast are distinct and operate independently. Anatomical research indicates that breast lymphatics form a subareolar plexus before draining into the central group of axillary lymph nodes.^25^ Meanwhile, the lymphatics from the upper limb follow a main medial pathway, joining axillary nodes and converging into ARM nodes. The separation of these pathways has been demonstrated in animal models.^26^

The ARM node is most commonly found above the second intercostobrachial nerve, although its exact location can vary.^11,15,27^ In this study, most ARM nodes were located in the upper lateral axilla (68.5%). This area is between the axillary vein and second intercostobrachial nerve, and close to the anterior edge of the latissimus dorsi muscle, which is consistent with established anatomical patterns and findings from prior studies.^4,11,28^ Nonetheless, drainage to the upper inner axilla was seen in 20.7% of patients, underscoring the variability in lymphatic anatomy.

Traditional mapping techniques include blue dye and radioisotopes, with blue dye being most commonly used. Early studies that utilized blue dye reported relatively low detection rates (61–71%) and limited visualization of lymphatics. Additionally, blue dye had drawbacks of prolonged skin staining at injection site and risk of hypersensitivity reactions. To enhance ARM node detection and avoid these issues, use of radioisotope with technetium-99m and lymphoscintigraphy has been studied. However, while it can improve node identification, it does not allow for direct visualization of lymphatic vessels, which is an essential aspect of ARM.^5,10,22^ Indocyanine green fluorescence is relatively new and less commonly used. In this present study, the ICG fluorescence detection rate of 95% lies at the higher end of previously reported visualization rates (61–95%).^5,10,22^ Indocyanine green has a favorable safety profile, with no adverse reactions observed in this study.^29–31^ Compared with lymphoscintigraphy, ICG fluorescence is intuitive to use and provides real-time feedback, making it an attractive alternative.^15,29^ Its real-time imaging capability enhances visualization of lymphatic flow and node identification and can also support microsurgical procedures, such as lymphaticovenous anastomosis.^6–15^

Initial studies proposed that ARM nodes are typically spared from metastatic disease and that preserving them may reduce lymphedema rates. However, more recent data, including this present study, challenge this assumption.^11,32^ This current study observed that 18.9% of ARM nodes had metastatic disease, raising concerns about the oncological safety of preserving ARM nodes. Previous studies have also demonstrated overlap between ARM and SLNs, and alternative lymphatic pathways have been observed, suggesting a more complex drainage system than initially believed.^4,11,30^

Notably, neoadjuvant chemotherapy did not significantly impact ARM node metastasis in this study’s cohort. While some prior studies reported reduced metastatic rates in ARM nodes of patients who received neoadjuvant chemotherapy, likely owing to nodal downstaging, the present findings align with more recent studies that show no such effect.^11,33^ This discrepancy may reflect the higher nodal burden among patients in this current study.

Although conservation of upper limb SLNs is not recommended owing to the significant occurrence of metastatic ARM nodes, preservation of ARM nodes could potentially be considered in carefully selected patients with minimal axillary involvement identified through preoperative clinical assessment or imaging. To date, there is a paucity of studies evaluating predictors for metastatic ARM nodes.^11,29,34^ Determining predictors of ARM node metastasis may support the development of clinical guidelines for selective preservation of arm-draining nodes during ALND.

Consistent with previous studies, this study found that larger tumor size and greater nodal burden were independent predictors of ARM node metastasis.^12,21–23,32,35^ In cases of more advanced axillary disease, ARM nodes were more often involved, which could be linked to the natural progression of the disease.^36^ No association was found between the location of the ARM node and likelihood of metastasis. Other variables, such as tumor grade, receptor status, Ki67 index, and completion of neoadjuvant chemotherapy, did not significantly differ between patients that had ARM node metastasis and those that did not. These findings reinforce that ARM node preservation should not be performed routinely and may only be appropriate in select low-risk patients identified through careful preoperative assessment. Some studies suggest that ARM node preservation may be oncologically safe in selected patients, such as those undergoing completion ALND after a positive SLNB, particularly if the ARM node appears benign intraoperatively. However, this remains a debated topic particularly with limited data on recurrence rates in this context.^13,22,35^

### Limitations

This study is limited by its single-center design, which may impact the generalizability of findings, particularly given variability in surgical technique and near-infrared imaging technology across institutions. Additionally, this study did not investigate the crossover rate between breast and arm SLNs, because all patients underwent ALND. Prior studies have shown that although most SLNs were different from ARM nodes, there were some cases where the SLN was the same as the ARM node, indicating convergence of the two drainage pathways through the same node.^7,8^ This convergence highlights the complexity of lymphatic drainage and challenges in preserving an ARM node where the SLN and ARM drainage converge. Future randomized controlled trials are also needed to further assess the safety of the ARM technique and its potential to reduce lymphedema when ARM nodes are preserved during ALND in a selected population at low risk of ARM node metastases. Considering the results of this present study and available literature, the authors are preparing for a future trial. This would be in combination with plastic surgeons to evaluate immediate lymphaticovenous anastomosis with ICG fluorescence-guided ARM during ALND.

## Conclusions

This current study demonstrated that ARM nodes and lymphatics can be reliably identified using ICG fluorescence during ALND. Although ARM node preservation has been proposed as a strategy to reduce the risk of lymphedema, nearly 19% of patients were found to have metastatic involvement of the ARM node, particularly those with larger primary tumors and a higher axillary nodal burden. In light of these findings, ARM node conservation should be approached with caution. Instead, alternative strategies such as immediate lymphatic reconstruction may provide a safer means of minimizing lymphedema risk following axillary surgery as more data accrues in this area.

## Data Availability

The data that support the findings of this study are available on request from the corresponding author, CLN.
